# The Blood Pressure in the Left Ventricle and in the Aorta*Contained in the LXXIII Vol. of the Sitzunzsberichte der k. Acad. der Wissensch

**Published:** 1876-08

**Authors:** H. Gradle


### EXAMINER.

VOL. XXXIII.—AUGUST, 1876.—No. 8.

### Original Communications.

### THE BLOOD PRESSURE IN THE LEFT VENTRICLE AND IN THE AORTA.*

BY DR. H. GRADLE.

(From the Institute of Experimental Pathology, at Vienna.)

Some years ago Prof. Fick† made the interesting observation, that the blood pressure in the left ventricle of the dog's heart is less than in the aorta. He conducted the investigation by means of a long tube, which could be introduced through the carotid artery and aorta into the left ventricle, which was connected with a registering manometer, so that a graphic record was obtained alternately of the blood pressure in the aorta and in the left ventricle. "As soon as the tube —in open connection with the manometer—is pushed forward into the ventricle, the index of the manometer is observed to sink and record oscillations, of which the highest does not even reach the lowest pressure previously found in the

* Contained in the LXXIII Vol. of the Sitzunzsberichte der k. Acad. der Wissensch

† Jeber die Schwankungen des Blutdruckes in verschiedenen Abschnitten des Gefäss systems. Würzburger Verhandlungen, 1873.

aorta. On withdrawing the tube again into the aorta, the index records pulsations high above the summits of the ventricular waves." (loc. cit. p. 232.) Hereupon Fick resorted to a second manometer, connected with the crural artery, while the first was being introduced into the heart in the above manner. He thus assured himself that the tension in the crural artery was not altered by the introduction of the tube into the heart, and furthermore that it exceded the pressure existing in the left ventricle. After proving experimentally that this unexpected result was not due to inaccuracy of the manometers, Fick adds, as a further evidence of its correctness, that these differences in pressure only occur when the heart beats very rapidly (after section of the vagi). The difference in tension disappeared on irritation of one pneumo-gastric nerve.

As an explanation of this strange phenomenon, Fick assumes that the systolic force does not conduce as much to raise the pressure of the blood in the ventricle as to confer upon that fluid a great velocity. However, he does not deny his doubts as to the real existence of the entire phenomenon. Previously Chauveau and Marey had estimated the pressure in the aorta and ventricle of the horse, and found it about equal in both places.

In a later publication* Marey refers to these experiments, and adds that he does not hesitate to consider those differences found by Fick the result of too great an inertia of the manometers employed; in the ventricle the variations of pressure are so considerable and of such a rapidity that the manometer is not able to record them accurately.

I have tested the statements of Fick by means of his own, as well as two other methods. After opening the thorax of a curarized dog, maintained by artificial respiration, I either introduced a curved tube into the left ventricle, through a small opening made in the auricular process, or forced a sharp canula directly through the walls of the ventricle into its cavity. In some of the latter instances the heart suddenly

* Physiologie Expérimentale. Travaux du Laboratoire de M. Marey. Paris, 1875, p. 38, note.

ceased to beat, but in most cases the procedure was well borne, and I could even with impunity enter the canula a second time, after having withdrawn it—the wound giving rise to but little hemorrhage. The tube or canula being connected with a mercurial manomenter, while a second instrument communicated with the carotid artery; I thus obtained on the paper of the kymographion the simultaneous graphic record of aortic and ventricular tension.

Having convinced myself of the correctness of Fick's statements, I sought to ascertain whether the conditions of the phenomenon are of a purely mechanical nature. A dead heart was filled with water from a tube communicating with the left auricle, the aorta was prolonged by means of a rubber hose, the manometers connected as in a living animal and the systolic contraction imitated by compression with the hand. As the result I obtained a graphic trace quite similar to those furnished by the living animal. Hereupon I constructed a model of the circulation to study the phenomenon at leisure. An ordinary rubber balloon, similar to that of Davidson's syringe, supplied with water from a reservoir with low pressure on the one side, communicated at the other end with a tube, which for the sake of convenience we will call aorta, while we may refer to the balloon as the heart of the system, and to its mechanical compression as the systole, corresponding to the bicuspid and semi-lunar valves; the balloon was also supplied with a valve at each end, opening with the current. The balloon as well as the aorta communicated besides with lateral tubes, which could either be prolonged by means of perpendicular glass tubes, the height of water in which indicated the pressure directly, or which could be connected with mercurial or Fick's manometers, for the purpose of registering the pressure on the paper of the kymographion.

The experimental conditions in this system are most readily comprehended, when the aorta is closed at its terminal end; in this case the results to be described are the same whether the aorta consists of an elastic or an inelastic tube, as glass. The efflux of water being thus prevented, the fluid rises in both manometers to a height equal to the height of the reservoir, which being the lowest pressure attainable under the circumstances, we will call zero. On compressing the balloon a number of times the following is the result: The first systole raised the fluid to a certain height, equal in both manometers; on its cessation the pressure returns to zero in the heart, while the semi-lunar valves maintain the column of water in the aortic manometer at the point reached during the systole. The next systole, however, having but the same effect on the cardiac pressure as the first, lifts the column of water in the aortic manometer to a height exceeding the level of the fluid in the cardiac manometer itself, so that with each successive systole the difference between the cardiac and aortic pressure increases in favor of the latter. While the terminal end of the aorta is closed, the duration of the interval between the systoles is, of course, of no consequence as to the result. However, as the difference existing between the heights of the fluid in the two manometers augments, the additional increase of aortic tension becomes less and less with each following systole. The apparent contradiction, that the semi-lunar valves are opened by a systole, while the pressure in the aorta exceeds by far that in the heart itself, loses its obscurity on varying the rapidity of the systole. If the compression of the balloon is performed very slowly, the water in the aortic manometer begins to rise only when the same height has been reached in the cardiac manometer, and in this case it does not exceed the level in the latter. But as the balloon is compressed more and more rapidly a limit is formed, beyond which the difference between the two manometers begins to appear. In short, as the celerity of the systole increases, the difference between the cardiac and aortic pressure produced by the same augments. But since the semi-lunar valves can be opened only by a force exceeding the pressure exerted upon its aortic surface, we are safe in maintaining that the pressure indicated by the manometer communicating with the heart is not a full measure of the systolic force. In other words, the result of the systole is not only the augmented cardiac tension, but also the momentum conveyed to the fluid.

If the water is allowed to flow from the terminal end of the aorta, the conditions are somewhat altered, though the above considerations are still applicable. In case the entire mass of water, emptied into the aorta during the systole, is permitted to escape during the diastole, the above difference in tension, though produced during the systole, speedily disappears. But if the open end of the aorta is obstructed beyond this limit—or, what amounts to the same thing, if the interval between the systoles is correspondingly shortened—each following systole results in an augmentation of aortic tension on the same principle as when the aortic outlet is entirely closed. But in this case, also, the augmentation of aortic pressure becomes less and less with each following systole, until finally every systole raises the aortic pressure merely as much as it had fallen during the preceeding interval; so that the water in the aortic manometer pulsates at a fixed height, which however far exceeds the level of the fluid in the cardiac manometer. The difference between the cardiac and aorta manometers of a system imitating the arterial circulation therefore depends on:

I. The force of the systole.
II. The celerity of the systole.
III. The duration of the interval.
IV. The resistance to the current.

The possibility of a difference in the height of the fluid, or, in other words, the pressure in the two manometers, depends, of course, on the presence of the valve representing the semilunar valves, since on its removal the equilibrium is at once restored, and no further accumulation of aortic tension can be effected.

The conditions of pressure during life will be most readily appreciated by reference to the following table:

CURARIZED DOG—BOTH VAGI DIVIDED.

| Time. | Oscillations of Ventricle pressure. | Oscillations of Aortic pressure. | Difference between the maximum pressure in the ventricie and aorta. |
|---|---|---|---|
| | 48–74 mm. Hg. | 100—104 | 30 mm. |
| | ARTIFICIAL RESPIRATION SUSPENDED. | | |
| 12 Sec. | 70—104 | 160—164 | 60 |
| 12 " | 70—104 | 164—168 | 64 |
| 12 " | 62— 98 | 146—150 | 52 |
| 12 " | 60— 94 | 148—152 | 58 |
| 12 " | 62— 98 | 164—168 | 70 |
| 12 " | 72—108 | 180—186 | 78 |
| 12 " | 82—120 | 204—210 | 90 |
| 12 " | 88—126 | 220—228 | 102 |
| 12 " | 96—138 | 240—244 | 106 |
| | ARTIFICIAL RESPIRATION RESUMED. | | |
| 12 " | 120—162 | 234—242 | 80 |
| 20 " | 78—116 | 154—162 | 46 |

From the result of this experiment, as well as many others, we learn that the oscillations of pressure in the left ventricle are much greater than those occurring in the aorta; while, however, the minimum of tension in the latter vessel far exceeds the maximum pressure found in the heart itself. The comparison of the different records shows, that—conforming with the results found on the models—the difference increases with the force of the systoles; in other words, the higher the summit of the ventricular wave the greater will be the corresponding increase of the aortic pressure. Further on, the difference will rise with the resistance to the current in the arteries; on constricting the systemic arterioles by asphyxiating the animal the aortic pressure increases more than the ventricular tension. The celerity of the systole, which is of such influence in the model, is a factor that we cannot vary at liberty during life. Lastly by prolonging the interval between the systoles by irritation of the vagus we permit more blood to pass the capillaries, and hence the aortic pressure will be lower at the beginning of each systole, the longer the duration of the diastole. This result, however, can be masked by simultaneous augmentation of the resistance to the passage of the blood in the smaller vessels; and thus, while during asphyxia the pulse becomes much less frequent (when the vagi are intact), the difference in pressure increases, nevertheless, on account of the contraction of the arterioles.

I have attempted the destruction of the semi-lunar valves, in the living animal, by introducing a long sound through the right carotid artery, and in pushing it forward, perforating the pockets of the valve, while guided by the finger placed on the origin of the aorta. In two cases, in which the autopsy proved the destruction of two of the pockets, the cardiac trace approached that of the aortic pressure, though there was still some difference both in the height of the waves and in the absolute pressure. In a third dog, however, whose valve I succeeded in destroying entirely, the record of the ventricular waves was the exact *fac-simile* of the aortic pulsations in all details.

These observations on the circulation during life, as well as on a model imitating it, hence lead to the conclusions, that normally the blood pressure in the aorta exceeds that of the left ventricle, and that this depends on the momentum of the blood in the ventricular cavity, which is not registered by a manometer communicating with the heart. Finally, that the accumulation of tension in the aorta is possible only when the restoration of equilibrium is prevented by the action of the semi-lunar valves.

